# Who are the male sexual partners of adolescent girls and young women? Comparative analysis of population data in three settings prior to DREAMS roll-out

**DOI:** 10.1371/journal.pone.0198783

**Published:** 2018-09-28

**Authors:** Aoife M. Doyle, Sian Floyd, Kathy Baisley, Benedict Orindi, Daniel Kwaro, Thandiwe N. Mthiyane, Sheru Muuo, Maryam Shahmanesh, Abdhalah Ziraba, Isolde Birdthistle

**Affiliations:** 1 MRC Tropical Epidemiology Group, Department of Infectious Diseases, London School of Hygiene & Tropical Medicine, London, United Kingdom; 2 African Health Research Institute, KwaZulu- Natal, South Africa; 3 African Population and Health Research Center, Nairobi, Kenya; 4 Katholieke Universiteit Leuven, Leuven, Belgium; 5 Kenya Medical Research Institute, Gem, Siaya county, Kenya; 6 University College London, London, United Kingdom; 7 Department of Population Health, London School of Hygiene & Tropical Medicine, London, United Kingdom; Institute of Tropical Medicine, Antwerp, BELGIUM

## Abstract

**Background:**

The DREAMS (Determined Resilient Empowered AIDS-free, Mentored and Safe) Partnership aims to reduce HIV incidence among adolescent girls and young women (AGYW,15-24y) with a core package of evidence-based interventions. Some interventions, including voluntary HIV counselling and testing and circumcision, will be targeted at the male sexual partners of AGYW. A priority of DREAMS is to characterise the male partners for effective targeting.

**Methods:**

Using population-based data (2010–2015) in three DREAMS impact evaluation settings in Kenya and South Africa, we describe the demographic characteristics and sexual behaviour of male partners reported by AGYW, and the characteristics of males who report sexual activity with AGYW.

**Results:**

In all settings, over 90% of recent male partners reported by AGYW were aged <35 years. Median ages of spousal and non-spousal partners were 29 and 23 years respectively in uMkhanyakude (rural South Africa) and 21 and 20 years respectively in Nairobi (urban Kenya). Most males reporting an AGYW partner had never been married (89%) and many were in school (39%). Most male partners reported only 1 AGYW partner in the past year; in Gem (rural Kenya) and Nairobi 25%-29% reported 2+(AGYW or older female) partners. Concurrent partners were reported by 16% of male partners in Gem and 3–4% in uMkhanyakude. Two thirds of male partners in Gem reported testing for HIV in the past 6 months and under half in uMkhanyakude reported testing for HIV in the past year. Almost all (96%) partners in Nairobi were circumcised, compared to 45% in Gem and 43% in uMkhanyakude.

**Conclusions:**

With almost all AGYW’s sexual partners aged 15–34 years, this is an appropriate target group for DREAMS interventions. Encouraging young men to reduce their number of partners and concurrency, and uptake prevention and treatment services such as HIV testing, circumcision and ART is crucial in the effort to reduce HIV among both AGYW and young men.

## Introduction

HIV incidence is high among adolescent girls and young women (AGYW, 15–24 y) in Eastern and Southern Africa due to social, behavioural and biological factors [1], and AGYW are increasingly targeted by HIV prevention interventions. AGYW who have unprotected sexual intercourse with multiple sexual partners are at higher risk of acquiring HIV [2, 3]. However, any increased risk of infection associated with a higher number of sexual partners will depend on the characteristics of the sexual partners (e.g., number of partners’ partners, duration of infection) and behaviour within the partnerships (e.g., frequency of sex, consistency of condom use)[4–6]. Key phenomena of sexual networks that are believed to be important determinants of HIV transmission within a population include concurrency (partnerships overlapping in time), and age-mixing and spatial bridging (connections between sexual networks usually constrained within age groups or geographical location) [7]. Interest in the characterisation of sexual partners has led to the inclusion of detailed questions on sexual partners in many quantitative surveys in high HIV prevalence settings [8–10]. Qualitative methods have also been developed to identify partner types according to transactional and socio-economic needs [11].

Most research to date on the partners of AGYW has focused on the age difference between a young woman and her partner. However, evidence for an association between age-disparate sexual partnerships and HIV infection has been inconsistent. Studies in Uganda, Zimbabwe, South Africa, and Malawi have shown an increased risk of HIV associated with age-disparate partnerships [6, 12–15]. In contrast, two longitudinal cohort studies in South Africa did not find age-disparity to be associated with risk of HIV acquisition [2, 16]; with one study suggesting that age-disparity may be less of an issue now as more older men are on ART, younger females may be more selective when choosing older male partners, and the socio-economic differentials usually associated with sexual negotiation power may be less steep in their setting[2]. Another recent study in South Africa found younger male sexual partner age (<35 years), independent of the female respondent’s age, to be a risk factor for HIV acquisition[6], and a phylogenetic study showed that females <25 years were most likely infected by a male partner who was on average 8.7 years older[17]. Partner age is important as HIV prevalence is higher among older men than younger men, and greater age differences and certain age groups (e.g., men aged 25–34 years) are associated with both riskier sexual behaviour[15, 18] and lower levels of engagement with HIV prevention and treatment services[6, 17].

A limited number of studies have explored the risk associated with other characteristics of AGYW’s male partners. There is some evidence that a female partner’s HIV and STI risk is associated with the type of partnership (non-spousal, sex worker), sexual behaviour (inconsistent condom use, multiple partners) and violence within the partnership, and a male partner’s occupation (e.g., truck driver, mine worker), education level, travel habits, and alcohol use [19, 20].

The DREAMS (Determined Resilient Empowered AIDS-free, Mentored and Safe) core package includes interventions to decrease risk among the sexual partners of adolescent girls and young women. The core package includes the characterisation of male partners so as to target highly effective interventions such as HIV testing and linkage to treatment in those who are positive, and voluntary medical male circumcision (VMMC) for those who are negative [Ref: Saul et al in this Collection]. Condom promotion and distribution targets apply to both AGYW and their male partners [21]. As part of the baseline analyses for impact evaluations of DREAMS in three settings, we sought to identify the key characteristics of AGYW’s most recent sexual partner, including age disparity, voluntary male medical circumcision, knowledge of HIV status. Understanding which men are sexually involved with AGYW, in each evaluation setting prior to DREAMS implementation (2010–15), can help with the effective targeting of DREAMS interventions for male partners and will provide an important reference for future analysis of DREAMS’ impact. In this paper, we present a descriptive analysis of the characteristics of the male partners of AGYW living in two Kenyan and one South African DREAMS setting. We sought to answer this key research question: Who are the male sexual partners of adolescent girls and young women in three evaluation settings before the roll-out of DREAMS?

## Methods

### Study design and settings

The impact of DREAMS is being evaluated in several settings in sub-Saharan Africa including the health and demographic surveillance sites (HDSS) of Gem, Nairobi and uMkhanyakude. Gem sub-county, Siaya County, in southwest Kenya, is situated 74 km north-west of Kisumu. The HDSS platform of the Kenya Medical Research Institute (KEMRI) includes approximately 220,000 inhabitants in 385 predominantly rural villages who are surveyed over time to understand population demographics, burden of disease, and access to and utilisation of health services[22]. A nested cohort of approximately 15,000 individuals (from a random selection of one-quarter of the households in Gem), is followed longitudinally for more detailed sexual behavioural data via the Longitudinal Bio-behavioural Survey (LBBS). Nairobi is the capital city of Kenya with an estimated population of 3.1 million inhabitants in 2009 [23]. The Nairobi Urban Health DSS (NUHDSS), run by the African Population and Health Research Center (APHRC), was set up in 2002 to investigate the long-term impact of urban residence on social, economic, and health outcomes [24]. The NUHDSS covers a population of approximately 65 000 individuals in Korogocho and Viwandani, two large informal settlement areas in Nairobi. Korogocho has a more stable settled population, whereas, Viwandani has a younger, more mobile population. In 2006/7 the HIV prevalence in these slum areas was 6.0% among 15–19 year old females, 2.7% among 15–19 year old males, 8.4% among 20–24 year old females and 2.9% among 20–24 year old males [25]. UMkhanyakude is a district of the province of KwaZulu-Natal in South Africa. Since 2000, the African Health Research Institute (AHRI) (formerly the Africa Centre for Health and Population Studies) has been conducting household-based surveys to collect demographic data on a population of approximately 100,000 individuals. The surveyed population live primarily in rural areas though the area also includes an urban township and informal peri-urban settlements [26]. In addition to demographic surveillance, resident household members who are aged ≥15 years are invited to participate in an annual individual-level survey, which includes an interview on general health and sexual behaviour. In 2015, HIV prevalence among females was 11% and 34% among 15–19 year olds and 20–24 year olds respectively (Chimbindi et al, this Collection). Among males, HIV prevalence was 7.2% among 20–24 year olds and 27.6% among 25–29 year olds (Baisley et al, this Collection). One-third and two-thirds of AGYW aged 15–19 years and 20–24 years, respectively, and just below half of males aged 20–29 years had tested for HIV in the past 12 months (Chimbindi et al & Baisley et al, this Collection). Only 0.5% of AGYW were engaged or married (includes legal and traditional marriage [27]) (Chimbindi et al, this Collection).

### Population

In each of the above DREAMS settings, we identified the most recent relevant data collected prior to the implementation of DREAMS interventions (2016). Our populations of interest were (i) females aged 15–24 years (AGYW) and (ii) males of any age who reported that they had sexual activity with a female aged 15–24 years in the last 12 months. In all surveys, respondents reported on their sexual behaviour including first sexual experience, lifetime number of sexual partners, and the characteristics of their sexual partners and partnerships in the past 12 months. Data were collected through face-to-face interview with responses recorded directly into a tablet.

#### Gem

Data collected during round 2 of the longitudinal biobehavioural survey (2014/15) were analysed. All residents aged 13 years or older were eligible to participate in the survey. Data were available for 1207 females aged 15–19 years, 1046 females aged 20–24 years, and 5154 males aged 15–97 years.

#### Nairobi

Data collected during round 3 of the Transitions to Adulthood study (2010) [28] were analysed. Participants aged 12–24 years were randomly sampled from the NUHDSS database. Data were available on 457 females aged 15–19 years, 438 females aged 20–24 years, and 919 males (12–24 years).

#### uMkhanyakude

Data collected during the 2015 General Health Survey were analysed. All females aged 15–24 years and all males aged 15–95 years who were resident in the surveillance area were eligible and included. A total of 3154 AGYW (1881 aged 15–19 years, 1273 aged 20–24 years), and 4942 males were interviewed for at least one component of the survey. The sexual behaviour questionnaire was completed by 1616 (85.9%) females aged 15–19 years and 851 (66.8%) of females aged 20–24 years.

### Ethics and informed consent

The study protocols for the data collection in each setting received ethical and research clearance from national committees (Gem & Nairobi: Ethical review board of the Kenya Medical Research Institute (KEMRI) Number NON-SSC 271B; uMkhanyakude: Biomedical Research Ethics Committee of the University of KwaZuluNatal, South Africa, Reference Number BE290/16). Written informed consent was obtained from participants in all settings and parental/guardian consent was obtained for minors prior to participation. Original ethical clearances included permission to conduct descriptive analysis of collected surveillance data, to understand determinants of HIV risk, and thus covers the analyses included here.

### Measures

We described the characteristics of male partners reported by AGYW (first partner and up to three most recent partners in the past 12 months), and of males who reported sexual activity with an AGYW (based on reports of up to three most recent partners in the past 12 months).

In Nairobi, respondents were asked to report the characteristics of their last three sexual partners regardless of when the relationship started or ended. This analysis included the sub-set of partners who had sex with the AGYW respondent in the 12 months prior to the survey. In Gem and uMkhanyakude, respondents were asked to report the characteristics of their most recent partners within the 12 months prior to the survey.

In Nairobi and uMkhanyakude, the exact age of the sexual partner was recorded whereas in Gem only the relative age (older/same age/younger) and the number of years older/younger (<5 yrs, 5–10 yrs, 10+ yrs, don’t know) were recorded. In Gem, the analysis is therefore presented for three groups of men (based on their own age and the relative age of their partner):

1. Men of any age who reported at least one AGYW partner in the past 12m;
2. Men aged 20-34y who *possibly* had an AGYW partner in the past 12m; and
3. Men aged 35+y who *possibly* had an AGYW partner in the past 12m.

Results for the three groups of men are presented in the results tables but the accompanying text focuses on (i) the male partners who report an AGYW partner. It is difficult to compare the data on age of partners from different settings given the challenge categorising partner age in Gem, and the interview of only males aged 12–24 years in Nairobi. Furthermore, in Nairobi respondents were asked for the age of their partner when they first had sex, whereas, in the other two settings the current age of the partner was recorded.

Unless otherwise specified, the measures are based on the last three partners that the respondent had in the past 12 months. The partnership pattern measure was created using reports of sexual activity (ever, in the past 12 months) and the reported number of partners in the past 12 months. In Gem, respondents reporting multiple partnerships were further divided into those who reported only one ongoing partnership at the time of the survey and those who reported more than one ongoing partnership at the time of the survey. Respondents were categorised as having concurrent sexual partners if they reported that they had two or more current partners when asked ‘How many sexual partners do you currently have?’. In uMkhanyakude, respondents reporting multiple partnerships were divided into those with and without overlapping partnerships at some point in the past 12 months based on the reported date of first and last sex with each partner. Respondents were categorised as having concurrent sexual partners if they reported that they were in two or more relationships when asked ‘How many relationships are you in at the moment?’. The available data for Nairobi did not allow us to calculate a measure of concurrency. The characteristics of male partners in the past 12 months reported by AGYW are based on a dataset of all partners with each of the respondent’s partners represented as an individual record. To present similar categories across settings, response categories were occasionally grouped together. Where a measure was missing for <1% of the respondents, the respondents with missing values were excluded from that measure. All other missing values are presented in the tables and/or described in footnotes.

### Statistical analysis

Reported characteristics of partners and partnerships were summarised by gender and age group of the respondent, i.e., female reports of their own behaviour and male reports of their female partners (in 15–19, 20–24, and 15–24 year age groups). In the text, unless there are important differences between older and younger AGYW and/or their male partners, we discuss the data for all AGYW (15–24 years).

## Results

### Demographic profile of men reporting an AGYW partner

Of the 5154 male respondents (age 15+) in rural Kenya (Gem), 10.3% reported an AGYW partner in the 12 months prior to the survey. There were an additional 10.7% males aged 20–34 years and 5.8% males aged 35+ years who possibly had an AGYW partner in the past 12 months. Among the 919 males aged 12–24 years in urban Kenya (Nairobi), 20.1% reported an AGYW partner in the past 12 months. In rural South Africa (uMkhanyakude), of the 2959 male respondents (age 15+) who agreed to the sexual behaviour questionnaire, 23.6% reported an AGYW partner in the past 12 months. (Table 1).

**Table 1:** Demographic characteristics of males who report a partner aged 15–24 years in the past 12 months (based on last 3 partners, source: male interviews).

**A. Gem**								
	**All men interviewed**		**Men aged 15–34 yr who report that at least one partner was aged 15–24 yrs in the past 12 months**		**Men aged 20–34 yr who had at least one partner possibly aged 15–24 yrs in the past 12 months**		**Men aged 35+yr who had at least one partner possibly aged 15–24 yrs in the past 12 months**	
**N**		**5154**		**530**		**553**		**301**
	n	%	n	%	n	%	n	%
**Age**								
15–19	1221	23.7	175	33	0	0	0	0
20–24	794	15.4	309	58.3	202	36.5	0	0
25–34	976	18.9	46	8.7	351	63.5	0	0
35–44	580	11.3	0	0	0	0	57	18.9
45–54	443	8.6	0	0	0	0	71	23.6
55–64	467	9.1	0	0	0	0	87	28.9
65+	673	13.1	0	0	0	0	86	28.6
**Highest education level reached**								
*Not currently in school*								
None	198	3.9	2	0.4	5	0.9	21	7.0
Primary	2702	52.5	269	50.9	363	65.6	190	63.1
Secondary	807	15.7	75	14.2	111	20.1	69	22.9
Tertiary	162	3.2	15	2.9	17	3.1	21	7.0
*Currently in school*								
Primary	775	15.1	66	12.5	7	1.3	0	0.0
Secondary	456	8.9	86	16.3	41	7.4	0	0.0
Tertiary	43	0.8	16	3.0	9	1.6	0	0.0
**Occupation**								
Professional, business owner, skilled labourer	561	10.9	59	11.2	112	20.3	54	17.9
Farmer/fisherman	2103	40.8	126	23.8	212	38.3	187	62.1
Small business (e.g. sell maize), unskilled labourer	735	14.3	108	20.4	136	24.6	42	14.0
Homemaker	6	0.1	1	0.2	1	0.2	0	0.0
student	1278	24.8	168	31.8	57	10.3	0	0.0
unemployed	373	7.2	57	10.8	28	5.1	12	4.0
Other	93	1.8	10	1.9	7	1.3	6	2.0
**B. Nairobi**								
	**All men interviewed**		**Men who report that they had at least one partner aged 15–19 yrs in the past 12 months**		**Men who report that they had at least one partner aged 20–24 yrs in the past 12 months**		**Men who report that they had at least one partner aged 15–24 yrs in the past 12 months**	
**N**		**919**		**147**		**38**		**185**
	n	%	n	%	n	%	n	%
**Age**								
12–14	103	11.2	1	0.7	0	0.0	1	0.5
15–19	470	51.1	60	40.8	2	5.3	62	33.5
20–24	346	37.7	86	58.5	36	94.7	122	65.9
**Highest education level reached**^1^								
*Not currently in school*								
None	3	1.1	0	0.0	0	0.0	0	0.0
Primary	138	50.7	17	23.0	2	22.2	19	10.2
Secondary	92	33.8	46	62.2	2	22.2	48	25.8
Tertiary	39	14.3	11	14.9	5	55.6	16	0.6
*Currently in school*								
Primary	313	48.4	40	54.8	16	53.3	56	30.1
Secondary	286	44.2	16	21.9	11	36.7	27	14.5
Tertiary	48	7.4	17	23.3	3	10.0	20	10.8
**Income generating activiities (IGA) in the past month**								
Unestablished own business	33	3.6	5	3.4	4	10.5	9	4.9
Established own business	17	1.8	7	4.8	2	5.3	9	4.9
Informal casual	88	9.6	29	19.7	6	15.8	35	18.9
Informal salaried	25	2.7	9	6.1	4	10.5	13	7.0
Formal salaried	24	2.6	7	4.8	4	10.5	11	5.9
Formal casual	36	3.9	6	1.4	5	13.2	11	5.9
Urban agriculture	2	0.2	0	0.0	0	0.0	0	0.0
Didn’t engage in IGA in past month	672	73.1	78	53.1	11	29.0	89	48.1
Missing	22	2.4	5	3.4	2	5.3	7	3.8
**C. uMkhanyakude**^2^								
			**Men who report that they had a partner aged 15–19 yrs in the past 12 months**		**Males who report that they had a partner aged 20–24 yrs in the past 12 months**		**Males who report that they had a partner aged 15–24 yrs in the past 12 months**	
**N**				**327**		**374**		**699**
			n	%	n	%	n	%
**Age**								
12–14			NA	NA	NA	NA	NA	NA
15–19			189	57.8	11	2.9	200	28.6
20–24			127	38.8	220	58.8	345	49.4
25–34			11	3.4	137	36.6	148	21.2
35–44			0	0.0	5	1.3	5	0.7
45–54			0	0.0	1	0.3	1	0.1
55–64			0	0.0	0	0.0	0	0.0
65+			0	0.0	0	0.0	0	0.0
**Residence**								
Urban			16	4.9	24	6.4	40	5.7
Peri-urban			109	33.3	145	38.8	254	36.3
Rural			202	61.8	205	54.8	405	57.9
**Highest education level reached**								
*Not currently in school*								
None			0	0.0	1	0.3	1	0.1
Primary			14	4.4	18	5.0	32	4.7
Secondary			93	29.3	268	74.2	359	53.1
Tertiary			1	0.3	4	1.1	5	0.7
*Currently in school*								
Primary			7	2.2	1	0.3	8	1.2
Secondary			202	63.7	69	19.1	271	40.1
Tertiary			0	0.0	0	0.0	0	0.0
**Occupation**								
Unemployed			210	88.6	257	70.2	465	77.4
Part time			6	2.5	13	3.6	19	3.2
Full time			21	8.9	96	26.2	117	19.5

Note: missing values not presented in the table. Percentages use available data as denominator.

^**1**^ Nairobi: Education status was calculated based on the highest school year that was completed. Respondent may have attended but not completed a higher school year.

^**2**^ uMkhanyakude: Residence, education status, and occupation measured in the household survey (proxy respondent).

The majority of males who reported AGYW partners were aged 15–34 years (Table 1, S1–S6 Tables), as described in further detail below (Partner age matrices). The proportion of male partners who report having reached secondary school or higher varied between settings (Gem 36.2%; Nairobi 40.3%; uMkhanyakude 93.2%). In all settings, a considerable proportion of male partners were still in school (Gem 31.8%; Nairobi 55.4%; uMkhanyakude 40.5%). In Gem, 10.8% of male partners were unemployed, whereas in uMkhanyakude 77.4% of male partners aged 18+ years were unemployed. In Nairobi, 48.1% of male partners did not engage in an income generating activity in the month prior to the survey (Table 1).

### Demographic profile and sexual behaviour of AGYW

AGYW in Gem lived predominantly in rural areas and all the AGYW in Nairobi lived in urban areas. Two thirds of the uMkhanyakude AGYW were living in rural areas, 32% living in peri-urban areas, and the remaining 6% living in urban areas. The proportion of AGYW who had reached secondary school or higher varied greatly between settings (Gem 30.3%; Nairobi 52.2%; uMkhanyakude 94.9%). In all settings, a considerable proportion of AGYW aged 15–19 years were not currently in school (Gem 31.2%; Nairobi 17.3%; uMkhanyakude 15.9%) (Table 2). In Gem, 22.5% of AGYW aged 20–24 years reported that they were unemployed, and in Nairobi, two-thirds of AGYW aged 20–24 years reported no income generating activity in the previous month (data not shown). In uMkhanyakude, 93% of AGYW aged 18–24 years were unemployed with many still in school (Chimbindi et al in this collection).

**Table 2:** Marital status, education and sexual behaviour reported by AGYW.

**A. Gem**	**Age of AGYW**					
	**15–19 yrs**		**20–24 yrs**		**15–24 yrs**	
**N**		**1207**		**1046**		**2253**
	**n**	**%**	**n**	**%**	**n**	**%**
**Highest education level reached**						
*Not currently in school*						
None	3	**0.3**	9	**0.9**	12	**0.5**
Primary	295	**24.5**	658	**63.0**	953	**42.4**
Secondary	74	**6.1**	210	**20.1**	284	**12.6**
Tertiary	4	**0.3**	11	**1.1**	15	**0.7**
*Currently in school*						
Primary	576	**47.8**	27	**2.6**	603	**26.8**
Secondary	252	**20.9**	107	**10.3**	359	**16.0**
Tertiary	1	**0.1**	22	**2.1**	23	**1.0**
**Marital status**						
Never married	998	**83.0**	390	**37.3**	1388	**61.7**
Previously married	9	**0.8**	33	**3.2**	42	**1.9**
Currently married	196	**16.3**	622	**59.5**	818	**36.4**
**Partnership pattern**						
Never had sex	705	**58.4**	108	**10.3**	813	**36.1**
No sex in past 12 months	130	**10.8**	134	**12.8**	264	**11.7**
Single partner in the past 12 months	344	**28.5**	774	**74.1**	1118	**49.7**
Multiple partners in the past 12 months- 1 ongoing partnership at the time of the survey	20	**1.7**	19	**1.8**	39	**1.7**
Multiple partners in the past 12 months- 2+ ongoing partnerships at the time of the survey	8	**0.7**	9	**0.9**	17	**0.8**
**Number of partners in the past 12 months**^1^						
median (IQR)		**1 (1,1)**		**1 (1,1)**		
1	344	**92.5**	774	**96.5**	1118	**95.2**
2	24	**6.5**	23	**2.9**	47	**4.0**
3+	4	**1.1**	4	**0.5**	8	**0.7**
Don’t know	0	**0.0**	1	**0.1**	1	**0.1**
**Current concurrency (report that currently have 2+ partners)** ^1^	6	**1.6**	8	**1**	14	**1.2**
**At least one partner had concurrent partners in past 12m**^1^	53	**14.3**	126	**15.7**	179	**15.3**
**At least one partner with new partners in past 12 months**^1^	61	**16.4**	133	**16.6**	194	**16.5**
**B. Nairobi**						
	**15–19 yrs**		**20–24 yrs**		**15–24 yrs**	
**N**		**457**		**438**		**895**
	n	%	**n**	**%**	**n**	**%**
**Highest education level reached**^2^						
*Not currently in school*						
None	1	**0.2**	6	**1.4**	7	**0.8**
Primary	44	**9.6**	189	**43.2**	233	**26.0**
Secondary	30	**6.6**	100	**22.8**	130	**14.5**
Tertiary	4	**0.9**	43	**9.8**	47	**5.3**
*Currently in school*						
Primary	182	**39.8**	6	**1.4**	188	**21.0**
Secondary	182	**39.8**	57	**13.0**	239	**26.7**
Tertiary	14	**3.1**	37	**8.4**	51	**5.7**
**Marital status**						
Never married	420	**91.9**	210	**48.0**	630	**70.4**
Previously married	4	**0.9**	28	**6.4**	32	**3.6**
Currently married	33	**7.2**	200	**45.7**	233	**26.0**
**Partnership pattern**						
Never had sex	**336**	**73.5**	81	**18.5**	417	**46.6**
No sex in past 12 months	**12**	**2.6**	68	**15.5**	80	**8.9**
Single partner in the past 12 months	**59**	**12.9**	104	**23.7**	163	**18.2**
Multiple partners in the past 12 months	**18**	**3.9**	75	**17.1**	93	**10.4**
Missing	**32**	**7.0**	110	**25.1**	142	**15.9**
**Number of partners in the past 12 months (among those who had sex in past 12mth)** ^1^						
median (IQR)	1 (1,2)		1 (1,2)			
1	59	**76.6**	104	**58.1**	163	**63.7**
2	12	**15.6**	40	**22.4**	52	**20.3**
3+	6	**7.8**	35	**19.6**	41	**16.0**
**C. uMkhanyakude**^3^						
	**15–19 yrs**		**20–24 yrs**		**15–24 yrs**	
N		**1881**		**1273**		**3145**
	**n**	**%**	**n**	**%**	**n**	**%**
**Highest education level reached**						
*Not currently in school*						
None	2	**0.1**	4	**0.3**	6	**0.2**
Primary	19	**1.0**	28	**2.2**	47	**1.5**
Secondary	273	**14.7**	816	**65.5**	1089	**35.1**
Tertiary	0	**0.0**	7	**0.6**	7	**0.2**
*Currently in school*						
Primary	101	**5.5**	4	**0.3**	105	**3.4**
Secondary	1458	**78.7**	386	**31.0**	1844	**59.5**
Tertiary	0	**0.0**	1	**0.1**	1	**0.0**
**Marital status**						
Never married	1819	**99.8**	1221	**98.9**	3040	**99.4**
Previously married	0	**0.0**	0	**0.0**	0	**0.0**
Engaged	4	**0.2**	5	**0.4**	9	**0.3**
Currently married	0	**0.0**	8	**0.6**	8	**0.3**
**Partnership pattern**						
Never had sex	1193	**73.8**	172	**20.2**	1365	**55.4**
No sex in past 12 months	9	**0.6**	6	**0.7**	15	**0.6**
Single partner in the past 12 months	406	**25.1**	664	**78.1**	1070	**43.4**
Multiple partners- no overlap in the past 12 months based on dates of first and last sex	5	**0.3**	4	**0.5**	9	**0.4**
Multiple partners- some overlap in the past 12 months based on dates of first and last sex	3	**0.2**	4	**0.5**	7	**0.3**
**Number of partners in the past 12 months (among those who had sex in past 12mth)** ^1^						
median (IQR)		**1 (1,1)**		**1 (1,1)**		
1	406	**98.1**	664	**98.8**	1070	**98.5**
2	7	**1.7**	8	**1.2**	15	**1.4**
3+	1	**0.2**	0	**0.0**	1	**0.1**
Don’t know	0	**0.0**	0	**0.0**	0	**0.0**
**Current concurrency**^1^	7	1.7	7	1.0	14	**1.3**

Note: missing values not presented in the table. Percentages use available data as denominator.

^**1**^ Among those who reported sex in the past 12 months

^**2**^Nairobi: Education status was calculated based on the highest school year that was completed. Respondent may have attended but not completed a higher school year.

^**3**^ uMkhanyakude: Residence and education status measured in the household survey (proxy respondent). Marital status measured among those who agreed to the individual general health survey. Sexual behaviour variables measured only among those who agreed to the individual sexual behaviour survey.

Very few (<1%) of AGYW in uMkhanyakude were currently married or engaged to be married. In contrast, 16.3% and 7.2% of 15–19 year olds and 59.5% and 45.7% of 20–24 year olds in Gem and Nairobi, respectively, were married or living as married (Table 2). In all settings, a considerable proportion of AGYW aged 15–19 years, and the majority of AGYW aged 20–24 years reported having ever had sex. Among AGYW who reported having had sex in the past 12 months, most reported having only one partner in that time, with few reporting three or more sexual partners (Gem 0.7%; Nairobi 16%; uMkhanyakude 0.1%). AGYW reported a median lifetime number of partners of 2 in Gem and 1 in Nairobi and uMkhanyakude (S7 Table).

Among those who reported two or more partnerships in the past year, less than half (Gem 30.4%; uMkhanyakude 43.8%) reported that they had concurrent (overlapping in time) partnerships. Less than 2% of AGYW who were sexually active in the past 12 months reported having concurrent partners at the time of the survey. In Gem, when AGYW were questioned about the behaviour of their recent partners, 15.3% reported that at least one of their partners had a concurrent partner in the past 12 months. A similar proportion reported that at least one of their male partners had new partners in the past 12 months (Table 2).

### Sexual behaviour among men who have AGYW partners

Most male partners had never been married (Gem 80.7%; Nairobi 77.3%; uMkhanyakude 99.3%). and reported only 1 partner in the past 12 months (Gem 71.1%; Nairobi 74.5%; uMkhanyakude 96.6%) (Table 3). Approximately, one in ten male partners in both Kenyan sites reported three or more partners in the past 12 months. In contrast, no male partners in uMkhanyakude reported three or more partners in the past year.

**Table 3:** Profile of males who report a partner aged 15–24 years in the past 12 months (based on last 3 partners, source: Male interviews).

**A. Gem**								
	**All men interviewed**		**Men aged 15–34 yr who report that at least one partner was aged 15–24 yr**		**Men aged 20–34 yr who report at least one partner who was possibly aged 15–24 yr**		**Men aged 35+yr who report at least one partner who was possibly aged 15–24 yr**	
**N**		**5154**		**530**		**553**		**301**
	n	%	n	%	n	%	n	%
**Marital status**								
Never married	2154	**41.9**	425	**80.7**	193	**35**	0	**0.0**
Previously married	299	**5.8**	6	**1.1**	15	**2.7**	10	**3.4**
Currently married	2689	**52.3**	96	**18.2**	344	**62.3**	288	**96.6**
**Partnership pattern**^1^								
Single partner in the past 12 months			377	**71.1**	412	**74.5**	193	**64.1**
Multiple partners in the past 12 months- 1 ongoing partnership at the time of the survey			87	**16.4**	78	**14.1**	15	**5.0**
Multiple partners in the past 12 months—2+ ongoing partnerships at the time of the survey			66	**12.5**	63	**11.4**	93	**30.9**
**Number of partners in the past 12 months**^1^								
median (IQR)	1 (1,1)		1 (1,2)		1 (1,2)		1 (1,2)	
1	2616	**80.0**	377	**71.1**	412	**74.5**	193	**64.1**
2	471	**14.4**	93	**17.6**	98	**17.7**	88	**29.2**
3+	172	**5.3**	57	**10.8**	41	**7.4**	19	**6.3**
Don’t know	12	**0.4**	3	**0.6**	2	**0.4**	1	**0.3**
**Current concurrency** ^1^^**,**^ ^2^			82	**15.5**	64	**11.6**	94	**31.3**
**At least one partner had concurrent partners in past 12 months**^1^			118	**22.3**	100	**18.1**	19	**6.3**
**At least one partner with new partners in past 12 months**^1^			123	**23.2**	107	**19.4**	23	**7.6**
**Tested for HIV in past 6 months**								
Yes	2765	**54.7**	348	**66.8**	335	**62**	156	**53.1**
No	2293	**45.3**	173	**33.2**	205	**38**	138	**46.9**
**Medically circumcised (self-reported)**								
Yes	1708	**33.2**	238	**44.9**	201	**36.4**	57	**18.9**
No	3434	**66.8**	292	**55.1**	351	**63.6**	244	**81.1**
**B. Nairobi**								
	**All men interviewed**		**Men who report that they had at least one partner aged 15–19 yrs**		**Men who report that they had at least one partner aged 20–24 yrs**		**Men who report that they had at least one partner aged 15–24 yrs**	
**N**		**919**		**147**		**38**		**185**
	n	%	n	%	n	%	n	%
**Marital status**								
Never married	824	89.7	119	81.0	24	63.2	143	77.3
Previously married	19	2.1	20	13.6	12	31.6	32	17.3
Currently married	76	8.3	8	5.4	2	5.3	10	5.4
**Number of partners in the past 12 months**^1^								
median (IQR)	1 (1,2)		1 (1,1)		1 (1,2)		1 (1,1)	
1	182	71.1	110	75.3	27	71.1	137	74.5
2	42	16.4	23	15.8	6	15.8	29	15.8
3+	32	12.5	13	8.9	5	13.2	18	9.8
**Medically circumcised (self-reported)**								
Yes	869	91.7	141	95.9	37	94.9	178	95.7
No	79	8.3	6	4.1	2	5.1	8	4.3
**C. uMkhanyakude**^4^^,^^5^								
			**Men who report that they had at least one partner aged 15–19 yrs**		**Men who report that they had at least one partner aged 20–24 yrs**		**Men who report that they had at least one partner aged 15–24 yrs**	
**N**				**327**		**374**		**699**
			n	%	n	%	n	%
**Marital status**^6^								
Never married			326	**99.7**	370	**98.9**	694	**99.3**
Previously married			0	**0.0**	0	**0.0**	0	**0.0**
Currently married			1	**0.3**	4	**1.1**	5	**0.7**
**Partnership pattern**								
Never had sex			0	**0.0**	0	**0.0**	0	**0.0**
No sex in past 12 months			0	**0.0**	0	**0.0**	0	**0.0**
Single partner in the past 12 months			317	**96.9**	358	**96**	675	**96.7**
Multiple partners- no overlap of partnership in the past 12 months based on dates of first and last sex			5	**1.5**	7	**1.9**	12	**1.7**
Multiple partners- some overlap of partnerships in the past 12 months based on timing of first and last sex with each partner			5	**1.5**	8	**2.1**	11	**1.6**
**Number of partners in the past 12 months**^1^								
median (IQR)			1(1,1)		1(1,1)		1(1,1)	
1			317	**96.9**	358	**95.7**	675	**96.6**
2			10	**3.1**	15	**4.0**	23	**3.3**
3+			0	**0.0**	0	**0.0**	0	**0.0**
Don’t know			0	**0.0**	1	**0.3**	1	**0.1**
**Current concurrency**^1^^**,**^^9^			14	**4.3**	19	**5.1**	31	**4.4**
**Tested for HIV in past 12 months**^7^								
Yes			145	**44.3**	181	**48.4**	324	**46.4**
No			182	**55.7**	193	**51.6**	375	**53.6**
**Medically circumcised (self-reported)**								
Yes			166	**51.2**	133	**36.3**	298	**43.3**
No			158	**48.8**	233	**63.7**	390	**56.7**
**HIV status**^8^								
Negative			262	**94.6**	226	**85.3**	486	**90.0**
Positive			15	**5.4**	39	**14.7**	54	**10.0**
*Unknown*			*50*	***(15*.*3)***	*109*	***(29*.*1)***	*159*	***(22*.*7)***

Note: missing values not presented in the table. Percentages use available data as denominator.

^**1**^ Among those who reported sex in the past 12 months

^**2**^ Gem: Based on response to question: How many sexual partners do you currently have?

^**3**^ Gem: Based on response to question asked of last 3 partners in the past 12 months: Is this relationship ongoing?

^**4**^ uMkhanyakude: Marital status measured among those who agreed to the individual general health survey. Sexual behaviour variables measured only among those who agreed to the individual sexual behaviour survey.

^**5**^uMkhanyakude: missing data on partner relationships for: N = 4 with AGYW partner age 15–19, and N = 6 for AGYW partner age 20–24; missing data for N = 4 AGYW aged 15–19 & N = 3 aged 20–24 who do not give info on relationship w/ partner(s)

^**6**^ uMkhanyakude: currently married includes those who are engaged

^**7**^ uMkhanyakude: Excludes testing in the DSS serosurvey since results are for research purposes only and are not returned to participant

^**8**^uMkhanyakude: HIV status from 2015 serosurvey. Unknown are those individuals who did not participate in the serosurvey in 2015; percentage is proportion who did not participate.

^9^ uMkhanyakude: Based on response to the question ‘How many relationships are you in at the moment?’

In Gem, 15.5% of males with an AGYW partner reported that they currently had at least two partners at the time of the survey (current concurrency). In contrast, 4.4% of male partners in uMkhanyakude reported having concurrent partners. In Gem, when questioned about their partners’ behaviour, 22.3% of male partners reported that at least one of their partners had a concurrent partner in the past 12 months. A similar proportion reported that at least one of their partners had new partners in the past 12 months.

In Gem, males who definitely had an AGYW partner and males aged 20–34 years who possibly had a AGYW partner reported similar partnership patterns. In contrast, males aged 35+ who possibly had a AGYW partner were more likely than the other groups to report multiple and overlapping partnerships. Reported concurrency at the time of the survey was 31% in this older group of male partners compared to 12–16% in the other two groups of male partners. Furthermore, the older male partners reported that a higher proportion of their concurrent partners were a mixture of spousal/regular and casual partners compared to the other two groups of men (14% vs. 2–3%). The older group of male partners, however, reported that a lower proportion of their female partners had concurrent partners or new partners in the past 12 months (6–7% vs. 18–23%) (Table 3).

### Service uptake and HIV status of men who have AGYW partners

In Gem, two thirds of male partners of AGYW had tested for HIV in the 6 months prior to the survey, whereas in uMkhanyakude less than half of male partners had tested for HIV in the past year. Almost all (96%) of male partners in Nairobi reported that they had had a medical circumcision, with levels lower in Gem (44.9%) and uMkhanyakude (43.3%). In uMkhanyakude, 5.4% and 14.7% of the male partners of AGYW aged 15–19 years and 20–24 years, respectively, were infected with HIV (Table 3).

### Characteristics of the first sexual partners of AGYW (Gem and Nairobi only)

Among AGYW in both Gem and Nairobi, most first sexual partners were boyfriends (85.9% & 57.3%) at the time of first sex. Few (10.1% and 11.3%, respectively) first partners were described as ‘spouse’ and 3.8% and 0.2% were reported to be casual acquaintances at the time of first sex. In Gem, one woman, and in Nairobi 12 women, reported that their first sex had been forced. In Nairobi, 9% of AGYW reported marrying their first partner. In both settings, most first partners were the same age or older, with 2% of AGYW in Gem reporting that their first sexual partner was more than 10 years older (Table 4).

**Table 4:** Characteristics of first male sexual partner (reported by females).

	Gem						Nairobi					
	Age of female respondent						Age of female respondent					
Characteristics of first sexual partner	15–19 years		20–24 years		15–24 years		15–19 years		20–24 years		15–24 years	
N		502		936		1438		121		357		478
	n	%	n	%	n	%	n	%	n	%	n	%
**Type of partner**												
Spouse	37	**7.4**	108	**11.5**	145	**10.1**	8	**6.6**	46	**12.9**	54	**11.3**
Girlfriend/boyfriend	440	**87.7**	792	**84.6**	1232	**85.7**	79	**65.3**	195	**54.6**	274	**57.3**
Casual Acquaintance/prostitute	21	**4.2**	34	**3.6**	55	**3.8**	0	**0.0**	1	**0.3**	1	**0.2**
Other	3	**0.6**	0	**0.0**	3	**0.2**	1	**0.8**	4	**1.1**	5	**1.0**
Missing	1	**0.2**	2	**0.2**	3	**0.2**	33	**27.3**	111	**31.1**	144	**30.1**
**Married first partner**												
Yes							2	**2.3**	28	**11.4**	30	**9.0**
No (includes live-in partner/boyfriend/casual)							86	**97.7**	218	**88.6**	304	**91.0**
**Relative age of partner**												
Same age	201	**40.0**	391	**41.9**	592	**41.2**	7	**5.8**	15	**4.2**	22	**4.6**
Older	280	**55.8**	500	**53.5**	780	**54.3**	77	**63.6**	212	**59.4**	289	**60.5**
Younger	4	**0.8**	11	**1.2**	15	**1.0**	5	**4.1**	16	**4.5**	21	**4.4**
Unknown (Gem)/ missing (Nairobi)	17	**3.4**	32	**3.4**	49	**3.4**	32	**26.4**	114	**31.9**	146	**30.5**
**Number of years older than respondent**												
Less than 5 years	209	**74.6**	351	**70.2**	560	**71.8**						
5–10 years	44	**15.7**	105	**21.0**	149	**19.1**						
More than 10 years	10	**3.6**	20	**4.0**	30	**3.8**						
Don’t know	17	**6.1**	24	**4.8**	41	**5.3**						

### Characteristics of male partners of AGYW

The type of partners reported by AGYW and their male partners are described in Table 5 and S7 Table. In summary, respondents in Gem and Nairobi reported primarily single spousal or regular partners, and multiple and overlapping spousal and regular partners. In uMkhanyakude, single partners were usually described as regular partners, and multiple and overlapping partnerships were with a mixture of casual and regular partners. Male partners of 15–19 year old AGYW compared to the partners of 20–24 year old AGYW reported a lower proportion of spousal partners in Gem (15.4% vs 50.6%), and, in uMkhanyakude, a higher proportion of casual partners (20.7% vs 8.2%).

**Table 5:** Characteristics of male partners reported by AGYW in the past 12 months (unit = partner).

**A. Gem**	**Type of partner**					
	**Spousal/ co-resident partner**		**Other regular**		**Casual**	
N (row %)	**801**	**(66.4%)**	**394**	**(32.7%)**	**11**	**(0.9%)**
	**n**	**%**	**n**	**%**	**n**	**%**
**Relative age of partner**						
Same age	144	18.0	105	26.7	2	18.2
Older	626	78.4	277	70.3	8	72.2
Younger	10	1.3	2	0.5	0	0.0
Not known	19	2.4	10	2.5	1	9.1
**Number of years older**						
Less than 5 years	335	53.5	218	79.0	3	37.5
5–10 years	199	31.8	43	15.6	3	37.5
More than 10 years	79	12.6	6	2.2	0	0.0
Don’t know / Missing	13	2.1	10	3.6	2	25.0
**Marital status of partner**						
Single	1	0.1	367	93.4	8	72.7
Married elsewhere	2	0.3	19	4.8	3	27.3
Previously married	7	0.9	4	1.0	0	0.0
Married to the respondent only	746	93.1	0	0.0	0	0.0
Married to the respondent and another wife	45	5.6	0	0.0	0	0.0
Don’t know	0	0.0	3	0.8	0	0.0
**Partner worked away from home (stayed overnight) in past 12 months**						
Yes	183	22.9	89	22.6	3	27.3
No	615	76.8	274	69.5	7	63.6
Don’t know/not applicable	3	0.4	31	7.9	1	9.1
**Has concurrent partners**						
Yes	76	9.5	49	12.4	3	27.3
I think so	31	3.9	25	6.4	1	9.1
No	388	48.4	135	34.3	3	27.3
Don’t know/refuse to answer	306	38.2	185	46.7	4	36.4
**Partner circumcised**						
No	564	70.4	131	33.3	7	63.6
Yes	206	25.7	228	57.9	4	36.4
Don’t know/ refuse to answer	31	3.9	35	8.9	0	0.0
**HIV status of partner**						
Known to be positive	58	7.3	5	1.3	0	0.0
Suspected to be positive	1	0.1	0	0.0	0	0.0
Known to be negative	452	57.1	222	57.4	4	36.4
Suspected to be negative	36	4.6	13	3.4	0	0.0
Unknown	244	30.9	147	38.0	7	63.6
**B. Nairobi**	**Type of partner**^1^					
	**Spouse**		**Boyfriend**		**Casual**	
N (row %)	56	(11.2%)	220	(43.9%)	3	(0.6%)
	**n**	**%**	**n**	**%**	**n**	**%**
**Relative age of partner**						
Same age	49	87.5	185	84.1	2	
Older	2	3.6	18	8.2	1	
Younger	4	7.1	16	7.3	0	
Not known	0	0.0	0	0.0	0	
Missing	1	1.8	1	0.5	0	
Median (IQR) age	21 (17,24)		20 (17,23)		20 (17,23)	
**Number of years older**						
Less than 5 years	23	46.9	117	63.2	1	
5–10 years	18	36.7	50	27.0	1	
More than 10 years	3	6.1	8	4.3	0	
Not known	5	10.2	10	5.4	0	
Missing	1	1.8	0	0.0	0	
**Residence**						
Same household	41	73.2	113	51.4	2	
Same town/village	0	0.0	3	1.4	0	
Other urban area	1	1.8	25	11.4	0	
Other rural area	6	10.7	64	29.1	1	
Missing	8	14.3	15	6.8	0	
**Level of concern that might get HIV/STI from this partner**						
Very concerned	15	26.8	118	53.6	1	
Somewhat concerned	4	7.1	21	9.6	1	
Not concerned	37	66.1	81	36.8	1	
**C. uMkhanyakude**						
	**Spousal**^2^		**Other regular**		**Casual**	
N (row %)	7	(0.6%)	993	(90.4%)	99	(9.0%)
	n	%	n	%	n	%
**Relative age of partner**						
Same age	0	0	123	12.4	19	19.2
Older	7	100	813	81.9	75	75.8
Younger	0	0	17	1.7	1	1
Not known	0	0	40	4	4	4
Median (IQR) age	29 (27–37)		23 (21–26)		23 (20–26)	
**Residence**						
In same local area	6	85.7	442	44.5	41	41.4
In another local area	1	14.3	546	55	57	57.6
Don’t know/missing	0	0	5	0.5	1	1

Note: missing values not presented in the table. Percentages use available data as denominator. Percentages are not presented for casual partners in Nairobi as the numbers were too small.

^**1**^Gem:’Spousal’ includes live-in partner; ‘casual’ includes commercial sex worker, fishmonger and other

^**2**^Nairobi: Type of partner missing for n = 222 partners

^**3**^ uMkhanyakude: Spousal partners include partners with whom the respondent is engaged to be married.

In all three settings, AGYW reported that the majority (67%+) of male partners were older (Table 5). In uMkhanyakude and Nairobi, where exact age of the partner was reported, the median age of male spousal partners was 29 years and 21 years respectively. The median age of other regular and casual male partners was 23 years in uMkhanyakude and 20 years in Nairobi. On average, AGYW reporting male partners were younger in Nairobi compared to uMkhanyakude, and in both settings AGYW were younger than their male partners. In Nairobi, the median age of AGYW reporting spousal/other regular partners was 18 years, and for AGYW reporting casual partners was 20 years. In uMkhanyakude, the median age of AGYW reporting spousal partners was 23 years, and for AGYW reporting other regular/casual partners 20 years.

Approximately a quarter of male partners in Gem had travelled overnight for work in the past 12 months. In Nairobi and uMkhanyakude, spousal partners were more likely to be living in the same local area compared to other regular and casual partners (Nairobi: spousal 73.2% (41), regular, 52.8% (113), casual 66.7% (2); uMkhanyakude: spousal 85.7% (6), regular 44.5% (442), casual 41.4% (41)).

In Gem, AGYW reported that 6% of their spousal partners were married to another wife, and 5% and 27% respectively of other regular and casual partners were married to someone else. Just over a third of casual partners were known or suspected to have concurrent partners compared to 13.4% of spousal partners and 18.8% of regular partners (Table 5). A similar proportion of each partner type was known or suspected to have acquired new partners in the past year (S8 Table). The proportion of partners who were circumcised varied according to partner type, with circumcision highest among regular partners (57.9%) and lowest among spousal partners (25.7%) (Table 5). Just over half of regular and casual partners at least sometimes gave money or gifts to the AGYW. AGYW always or sometimes gave money or gifts to ~10% of their regular and casual partners. AGYW reported that in 8.2% of their partnerships in the past 12 months, their partner had forced them to have sex at least once in the past 12 months (S8 Table).

In Gem, AGYW knew the HIV status of 64.4% and 58.7% of their spousal and regular partners, respectively, but only 36.4% of their casual partners. A higher proportion of spousal partners were known or suspected to be HIV positive compared to other types of partners (7% vs. 0–1%). Almost all (95%+) knew their partners’ status because their partner told them or they tested together. In Nairobi, AGYW were very or somewhat concerned that they might get HIV or an STI from approximately two thirds of their regular and casual partners but only one third of their spousal partners (Table 5).

### Partner age matrices

In all settings, almost all (90%+) of the partners reported by 15–24 year olds males were aged <25 years (S1, S3 and S5 Tables). In uMkhanyakude, the proportion of partners who were AGYW decreased as the male respondent’ age increased from 51.7% for 25–29 year olds males to 13.1% and 1.2% for 30–34 year old and 40–44 year old males respectively (S5 Table). In all settings, over 90% of the male partners reported by AGYW were aged <35 years (S2, S4 and S6 Tables). At least three quarters of male partners reported by 15–19 year old AGYW were <25 years of age (Gem 74.6%; Nairobi 86.0%; uMkhanyakude 91.1%). A lower proportion of the partners reported by 20–24 year old AGYW were aged <25 years (Gem 22.7%; Nairobi 69.3%; uMkhanyakude 45.0%).

## Discussion

Our analysis found that most partners of AGYW (including first partners) were young men under 35 years of age. Few male partners were married and many were still in school. HIV testing and circumcision uptake among partners of AGYW were relatively low and fell well short of national targets, except for Nairobi where reported circumcision was 96%. Many partners travelled overnight for work and/or were not living in the study area implying that, even when scaled-up, geographically-focused interventions like DREAMS are unlikely to reach all potential partners of AGYW (and may miss the higher risk partners). This highlights the importance for specific interventions for mobile populations. Partners of AGYW in Gem and Nairobi, rural and urban Kenya, reported relatively high levels of multiple and/or concurrent partners, and AGYW in Gem also reported that a relatively high proportion of their partners had concurrent partners. Taken together these findings emphasise the importance of understanding the risk within AGYW’s sexual networks as opposed to focusing only on the AGYW’s own reported behaviour.

This cross-setting comparison revealed some differences in the male partners of AGYW including higher levels of reporting of multiple and concurrent partners by male partners in the two Kenyan settings compared to uMkhanyakude, South Africa. Few females reported multiple partners in Gem and uMkhanyakude. Male partners in Gem were more likely to be described as spousal or regular partners, whereas in uMkhanyakude both AGYW and their male partners reported primarily non-spousal regular and casual partners. These reporting patterns are likely to reflect the social context with marriage, and hence spousal partners, being very rare in uMkhanyakude compared to Gem. The pattern of partnerships in Nairobi was unclear as challenges linking the individual and partnership datasets resulted in many missing values. For example, partnership loop data including detailed data on type of partner, age of partner etc. was missing for 222 (44.3%) partners reported by AGYW in Nairobi. The data appear to be missing in a random fashion but we cannot rule out the possibility of biased reporting. Furthermore, as only males <25 years were interviewed in Nairobi, we cannot describe partnerships between AGYW and older men in this context.

Some variation was observed in the characteristics of the different partner types. For example, spousal partners tended to be older, and a higher proportion of regular and casual partners compared to spousal partners were reported to be living away from the community. In Gem, where more detailed characterisation of partners was possible, a higher proportion of spousal partners were reported to be HIV positive compared to other partner types, and a higher proportion of casual partners were reported to be married to someone else, and to have concurrent partners. It is important to note, however, that respondents were more likely to know the HIV status of spousal compared to other types of partners; and in Nairobi, AGYW were more concerned about contracting HIV from a non-spousal partner (Table 5). Also, the categorisation of partners as spousal, regular or casual is over-simplistic and is likely to mask more subtle but important differences in the nature of of different relationships [10, 29].

We stratified our analysis of partnership patterns according to two age groups for AGYW. In general, patterns of sexual behaviour and reports of partners were similar between the two groups. As AGYW get older they are more likely to become sexually active and there is some suggestion in the data that a higher proportion of girls who first become sexually active at a younger age tend to engage in higher risk sexual practices. For example, in Kenya a higher proportion of sexually active 15–19 year olds reported multiple partners compared to sexually active 20–24 year olds. Early age at first sex has been shown elsewhere to be associated with higher risk sexual behaviour [30]. Alternatively, it is possible that older married AGYW may be more reluctant to report their non-spousal partners for fear of stigma and repercussions [29] or their behaviour might become lower-risk when they are older.

A strength of this study is the analysis of population based data from three settings in sub-Saharan Africa. We described sexual behaviour and partnership patterns from both the AGYW and male partner perspective, to better understand the wider sexual networks to which AGYW are linked. Comparing data across settings has shown the importance of understanding local networks and contexts, in identifying opportunities for HIV prevention. An unusually large variety of indicators were available to describe the sexual partners of AGYW, especially in Gem, and this allowed us to present a uniquely in-depth analysis of the characteristics of male partners in this setting. Many of the indicators that were only available in pre-2016 surveys for male partners in Gem are now included in questionnaires for all DREAMS evaluation sites, and will allow richer and more comprehensive cross-setting analysis in the future. The data presented are from surveys that aimed to capture representative samples, but not all AGYW are at high risk for HIV, and in this analysis we do not distinguish between partnerships of low/higher risk AGYW. Further analyses—based on risk profiles in each setting—could focus on the male partners of the “highest risk” AGYW.

A major limitation of sexual behaviour surveys is that respondents may underreport sensitive and socially undesirable behaviours including their number of sexual partnerships [31–34]. We believe that underreporting of the number of sexual partnerships is likely in all our study settings and that casual partnerships are more likely to have been underreported. The large difference between the reporting of multiple partnerships by males in the Kenyan settings and the South African setting is striking and may indicate a greater reluctance to report multiple sexual partners in uMkhanyakude. In the context of repeated surveys such as those that take place in a demographic surveillance area, there may be a tendency to report a lower number of partners to reduce the length of time of the interview [34]. Attempts to overcome these biases through the use of self-interview methods such as audio computer-assisted self interview (ACASI) have shown mixed evidence [33, 35]. Participation rates in repeated surveys in HDSS can also be low. In uMkhanyakude participation was lower overall among older males and females across all components of the survey, and most pronounced for the sexual behaviour questionnaire. Low participation rates would affect these findings if the partners of the AGYW who did not participate in the survey were different from the partners of the AGYW who did participate. For example, non-participants are often more mobile and at higher risk of HIV, and men can be more likely to refuse participation [36], [37].

In addition to potential underreporting of partnerships, the individual questions on each partner are subject to recall bias, and interviewer or respondent fatigue can also lead to missing data. For example, determining whether a respondent had concurrent partners or not often relies on accurate recording of the dates of first and last sex with each partner. In uMkhanyakude, 10 of the 12 male partners who reported multiple but not overlapping partnerships in the past 12 months (Table 3) had missing data on the timing of first or last sex and could have been misclassified. Similarly, in Gem, inconsistencies in the data on the timing of first and last sex with partners led us to use alternative measures of concurrency.

The survey in Gem chose to ask respondents about the relative age instead of the exact age of their sexual partners. This is a frequently used approach in settings where exact ages are difficult to obtain but prevented us from identifying all the males who had an AGYW partner. We created a category of men who possibly had an AGYW partner and split this into two different age groups (men <35 yrs, 35+yrs). Patterns of reported sexual behaviour and other demographic characteristics, such as occupation, varied considerably between these different age groups with, for example, older men reporting higher levels of multiple and concurrent partnerships. However, it is impossible to tell whether, for example, the older men (35+yrs) who reported some higher risk behaviours did have a AGYW partner. As mentioned above, categorising partners as ‘regular’ or ‘casual’ has its limitations as those categories can be interpreted differently and knowledge and recall of the characteristics of partners e.g. age and HIV status, is likely to differ between the different types of partners.

## Conclusions

The majority of the male partners of AGYW are aged 15–34 years confirming that this should be the target group for DREAMS interventions. Encouraging males in this age group to reduce their multiple and concurrent partners, and to take up prevention and treatment services such as HIV testing, circumcision and ART (Baisley et al, this Collection) is likely to reduce the risk of HIV infection among their AGYW partners [38]. At least 60% of the male partners reported by 15–24 year old AGYW were 20–29 years old. Partners in this age group are more likely than younger men to be HIV positive, and less likely than older men to be aware of their HIV status or to link into care and treatment to achieve viral suppression [39](Baisley et al, this collection). If resources are limited then prioritising preventative and treatment interventions for 20–29 year old males may be an effective strategy to break the cycle of HIV transmission [6, 40]. However, differences observed in the characteristics of partners and partnerships between DREAMS settings reflect variations in the cultural, socio-economic and urban/rural contexts, and emphasise the importance of understanding the local context when designing prevention programmes[41] (Saul et al, this Collection). Intervention approaches should be nuanced to take into account these varying contexts, and, ideally, should be developed around young men’s prevention needs for their own sake and not only as sexual partners of AGYW.

## Supporting information

S1 Dataset De-identified Nairobi/APHRC data. (XLSX)

S2 Dataset De-identified Gem/KEMRI data (individual data). (XLS)

S3 Dataset De-identified Gem/KEMRI data (partner data). (XLS)

S1 Table Gem partner age matrix: Male reports on female partners in the past 12 months. Data are row percentages. AGYW partners in dark grey shading. Possible AGYW partner in light grey shading.
(DOCX)

S2 Table Gem partner age matrix: Female reports on male partners in the past 12 months. Data are row percentages.
(DOCX)

S3 Table Nairobi partner age matrix: Male reports on female partners in the past 12 months. Data are row percentages.
(DOCX)

S4 Table Nairobi partner age matrix: Female reports on male partners in the past 12 months. Data are row percentages.
(DOCX)

S5 Table uMkhanyakude partner age matrix: Male reports on female partners in the past 12 months. Data are row percentages.
(DOCX)

S6 Table uMkhanyakude partner age matrix: Female reports on male partners in the past 12 months. Data are row percentages.
(DOCX)

S7 Table Gem/Nairobi/uMkhanyakude additional sexual behaviour data (AGYW and male partners). (DOCX)

S8 Table Gem additional data on characteristics of male partners. (DOCX)

## References

[1] WHO. Global accelerated action for the health of adolescents (AA-HA!): guidance to support country implementation. 2017.

[2] Harling G, Newell ML, Tanser F, Kawachi I, Subramanian SV, Barnighausen T. Do age-disparate relationships drive HIV incidence in young women? Evidence from a population cohort in rural KwaZulu-Natal, South Africa. J Acquir Immune Defic Syndr. 2014;66(4):443–51. Epub 2014/05/13. 24815854 10.1097/QAI.0000000000000198 PMC4097949

[3] UNAIDS. Get on the Fast-Track. The life-cycle approach to HIV. Geneva: UNAIDS, 2016.

[4] Tanser F, Barnighausen T, Hund L, Garnett GP, McGrath N, Newell ML. Effect of concurrent sexual partnerships on rate of new HIV infections in a high-prevalence, rural South African population: a cohort study. Lancet. 2011;378(9787):247–55. Epub 2011/07/19. 10.1016/S0140-6736(11)60779-4 21763937 PMC3141142

[5] Mah TL, Halperin DT. Concurrent sexual partnerships and the HIV epidemics in Africa: evidence to move forward. AIDS Behav. 2010;14(1):11–6; dicussion 34–7. Epub 2008/07/24. 10.1007/s10461-008-9433-x . 18648926

[6] Akullian A, Bershteyn A, Klein D, Vandormael A, Barnighausen T, Tanser F. Sexual partnership age pairings and risk of HIV acquisition in rural South Africa. Aids. 2017;31(12):1755–64. Epub 2017/06/08. 10.1097/QAD.0000000000001553 . 28590328 PMC5508850

[7] Helleringer S, Kohler HP. Sexual network structure and the spread of HIV in Africa: evidence from Likoma Island, Malawi. Aids. 2007;21(17):2323–32. Epub 2007/12/20. 10.1097/QAD.0b013e328285df98 . 18090281

[8] Program TD. DHS Model Questionnaire- Phase 7. Model Woman’s Questionnaire. 2015.

[9] Reniers G, Wamukoya M, Urassa M, Nyaguara A, Nakiyingi-Miiro J, Lutalo T, et al Data Resource Profile: Network for Analysing Longitudinal Population-based HIV/AIDS data on Africa (ALPHA Network). Int J Epidemiol. 2016;45(1):83–93. Epub 2016/03/13. 10.1093/ije/dyv343 . 26968480 PMC5823235

[10] Doyle AM, Plummer ML, Weiss HA, Changalucha J, Watson-Jones D, Hayes RJ, et al Concurrency and other sexual partnership patterns reported in a survey of young people in rural Northern Tanzania. PLoS One. 2017;12(8):e0182567 Epub 2017/08/25. 10.1371/journal.pone.0182567 . 28837686 PMC5570426

[11] Hallman Kelly P S, Jenkins Alison, Matee Neema, Paul Phillipo, Cerna-Turoff Ilan, Mrisho Fatma, and Bruce Judith. The ASERT method reveals a transactional—socio-emotional continuum of 13 sexual partner types among adolescent girls and young women in Tanzania. New York: Population Council, 2016.

[12] Biraro S, Ruzagira E, Kamali A, Whitworth J, Grosskurth H, Weiss HA. HIV-1 transmission within marriage in rural Uganda: a longitudinal study. PLoS One. 2013;8(2):e55060 Epub 2013/02/08. 10.1371/journal.pone.0055060 . 23390512 PMC3563659

[13] Schaefer R, Gregson S, Eaton JW, Mugurungi O, Rhead R, Takaruza A, et al Age-disparate relationships and HIV incidence in adolescent girls and young women: evidence from Zimbabwe. Aids. 2017;31(10):1461–70. Epub 2017/04/21. 10.1097/QAD.0000000000001506 . 28426534 PMC5457819

[14] Evan M, Risher K, Zungu N, Shisana O, Moyo S, Celentano DD, et al Age-disparate sex and HIV risk for young women from 2002 to 2012 in South Africa. J Int AIDS Soc. 2016;19(1):21310 Epub 2017/04/02. 10.7448/IAS.19.1.21310 . 28364564 PMC5384594

[15] Beauclair R, Helleringer S, Hens N, Delva W. Age differences between sexual partners, behavioural and demographic correlates, and HIV infection on Likoma Island, Malawi. Scientific reports. 2016;6:36121 Epub 2016/11/03. 10.1038/srep36121 . 27805053 PMC5090960

[16] Balkus JE, Nair G, Montgomery ET, Mishra A, Palanee-Phillips T, Ramjee G, et al Age-Disparate Partnerships and Risk of HIV-1 Acquisition Among South African Women Participating in the VOICE Trial. J Acquir Immune Defic Syndr. 2015;70(2):212–7. Epub 2015/06/08. 10.1097/QAI.0000000000000715 . 26049280 PMC4573287

[17] de Oliveira T, Kharsany AB, Graf T, Cawood C, Khanyile D, Grobler A, et al Transmission networks and risk of HIV infection in KwaZulu-Natal, South Africa: a community-wide phylogenetic study. The lancet HIV. 2017;4(1):e41–e50. Epub 2016/12/05. 10.1016/S2352-3018(16)30186-2 . 27914874 PMC5479933

[18] Maughan-Brown B, Evans M, George G. Sexual Behaviour of Men and Women within Age-Disparate Partnerships in South Africa: Implications for Young Women’s HIV Risk. PLoS One. 2016;11(8):e0159162 Epub 2016/08/16. 10.1371/journal.pone.0159162 . 27526116 PMC4985138

[19] Mathur S, Wei Y, Zhong X, Song X, Nalugoda F, Lutalo T, et al Partner Characteristics Associated With HIV Acquisition Among Youth in Rakai, Uganda. J Acquir Immune Defic Syndr. 2015;69(1):75–84. Epub 2015/01/27. 10.1097/QAI.0000000000000539 . 25622058 PMC4424095

[20] Swartzendruber A, Zenilman JM, Niccolai LM, Kershaw TS, Brown JL, Diclemente RJ, et al It takes 2: partner attributes associated with sexually transmitted infections among adolescents. Sex Transm Dis. 2013;40(5):372–8. Epub 2013/04/17. . 23588126 10.1097/OLQ.0b013e318283d2c9 PMC3894602

[21] PEPFAR. Preventing HIV in Adolescent Girls and Young Women. Guidance for PEPFAR Country Teams on the DREAMS Partnership. 2015 2015-03-09. Report No.

[22] Odhiambo FO, Laserson KF, Sewe M, Hamel MJ, Feikin DR, Adazu K, et al Profile: the KEMRI/CDC Health and Demographic Surveillance System—Western Kenya. Int J Epidemiol. 2012;41(4):977–87. Epub 2012/08/31. 10.1093/ije/dys108 . 22933646 PMC12083774

[23] Statistics KNBo. Population and household characteristics of Kenya, 2009 2009 [cited 2017 29 October]. http://kenya.opendataforafrica.org/jawneyf/population-and-household-characteristics-of-kenya-2009.

[24] Beguy D, Elung’ata P, Mberu B, Oduor C, Wamukoya M, Nganyi B, et al Health & Demographic Surveillance System Profile: The Nairobi Urban Health and Demographic Surveillance System (NUHDSS). Int J Epidemiol. 2015;44(2):462–71. Epub 2015/01/18. 10.1093/ije/dyu251 . 25596586

[25] Madise NJ, Ziraba AK, Inungu J, Khamadi SA, Ezeh A, Zulu EM, et al Are slum dwellers at heightened risk of HIV infection than other urban residents? Evidence from population-based HIV prevalence surveys in Kenya. Health Place. 2012;18(5):1144–52. Epub 2012/05/18. 10.1016/j.healthplace.2012.04.003 . 22591621 PMC3427858

[26] Tanser F, Hosegood V, Barnighausen T, Herbst K, Nyirenda M, Muhwava W, et al Cohort Profile: Africa Centre Demographic Information System (ACDIS) and population-based HIV survey. Int J Epidemiol. 2008;37(5):956–62. Epub 2007/11/14. 10.1093/ije/dym211 . 17998242 PMC2557060

[27] Hosegood V, McGrath N, Moultrie T. Dispensing with marriage: Marital and partnership trends in rural KwaZulu-Natal, South Africa 2000–2006. Demogr Res. 2009;20:279–312. Epub 2009/06/30. 10.4054/DemRes.2009.20.13 . 25729322 PMC4340557

[28] Beguy D, Mumah J, Gottschalk L. Unintended pregnancies among young women living in urban slums: evidence from a prospective study in Nairobi city, Kenya. PLoS One. 2014;9(7):e101034 Epub 2014/08/01. 10.1371/journal.pone.0101034 . 25080352 PMC4117474

[29] Plummer ML, Wight D. Young People’s Lives and Sexual Relationships in Rural Africa: Findings from a Large Qualitative Study in Tanzania: Lexington Books; 2011 8 16, 2011. 464 p.

[30] Pettifor AE, van der Straten A, Dunbar MS, Shiboski SC, Padian NS. Early age of first sex: a risk factor for HIV infection among women in Zimbabwe. Aids. 2004;18(10):1435–42. 15199320 10.1097/01.aids.0000131338.61042.b8

[31] Glynn JR, Carael M, Auvert B, Kahindo M, Chege J, Musonda R, et al Why do young women have a much higher prevalence of HIV than young men? A study in Kisumu, Kenya and Ndola, Zambia. Aids. 2001;15 Suppl 4:S51–60. yes. 11686466 10.1097/00002030-200108004-00006

[32] Nnko S, Boerma JT, Urassa M, Mwaluko G, Zaba B. Secretive females or swaggering males? An assessment of the quality of sexual partnership reporting in rural Tanzania. Soc Sci Med. 2004;59(2):299–310. yes. 10.1016/j.socscimed.2003.10.031 15110421

[33] Hewett PC, Mensch BS, Erulkar AS. Consistency in the reporting of sexual behaviour by adolescent girls in Kenya: a comparison of interviewing methods. Sex Transm Infect. 2004;80(suppl_2):ii43–8. 15572639 10.1136/sti.2004.013250 PMC1765856

[34] Harling G, Gumede D, Mutevedzi T, McGrath N, Seeley J, Pillay D, et al The impact of self-interviews on response patterns for sensitive topics: a randomized trial of electronic delivery methods for a sexual behaviour questionnaire in rural South Africa. BMC Med Res Methodol. 2017;17(1):125 Epub 2017/08/19. 10.1186/s12874-017-0403-8 . 28818053 PMC5561578

[35] Phillips AE, Gomez GB, Boily MC, Garnett GP. A systematic review and meta-analysis of quantitative interviewing tools to investigate self-reported HIV and STI associated behaviours in low- and middle-income countries. Int J Epidemiol. 2010;39(6):1541–55. Epub 2010/07/16. 10.1093/ije/dyq114 . 20630991

[36] McGrath N, Eaton JW, Newell ML, Hosegood V. Migration, sexual behaviour, and HIV risk: a general population cohort in rural South Africa. The lancet HIV. 2015;2(6):e252–e9. Epub 2015/08/19. 10.1016/S2352-3018(15)00045-4 . 26280016 PMC4533230

[37] Larmarange J, Mossong J, Barnighausen T, Newell ML. Participation dynamics in population-based longitudinal HIV surveillance in rural South Africa. PLoS One. 2015;10(4):e0123345 Epub 2015/04/16. 10.1371/journal.pone.0123345 . 25875851 PMC4395370

[38] Rositch AF, Cherutich P, Brentlinger P, Kiarie JN, Nduati R, Farquhar C. HIV infection and sexual partnerships and behaviour among adolescent girls in Nairobi, Kenya. Int J STD AIDS. 2012;23(7):468–74. Epub 2012/07/31. 10.1258/ijsa.2012.011361 . 22843999 PMC3571685

[39] Iwuji CC, Orne-Gliemann J, Larmarange J, Balestre E, Thiebaut R, Tanser F, et al Universal test and treat and the HIV epidemic in rural South Africa: a phase 4, open-label, community cluster randomised trial. The lancet HIV. 2018;5(3):e116–e25. Epub 2017/12/05. 10.1016/S2352-3018(17)30205-9 . 29199100

[40] Kripke K, Okello V, Maziya V, Benzerga W, Mirira M, Gold E, et al Voluntary Medical Male Circumcision for HIV Prevention in Swaziland: Modeling the Impact of Age Targeting. PLoS One. 2016;11(7):e0156776 Epub 2016/07/15. 10.1371/journal.pone.0156776 . 27410687 PMC4943626

[41] Napierala Mavedzenge S, Olson R, Doyle AM, Changalucha J, Ross DA. The epidemiology of HIV among young people in sub-Saharan Africa: know your local epidemic and its implications for prevention. J Adolesc Health. 2011;49(6):559–67. Epub 2011/11/22. 10.1016/j.jadohealth.2011.02.012 . 22098766

