# Supplementary material for: Who are the male sexual partners of adolescent girls and young women? Comparative analysis of population data in three settings prior to DREAMS roll-out
Source: PLoS One. 2018 Sep 28;13(9):e0198783. doi: 10.1371/journal.pone.0198783 (PMC6161870; doi:10.1371/journal.pone.0198783)
Supplement: S1 Table — Data are row percentages. AGYW partners in dark grey shading. Possible AGYW partner in light grey shading. (DOCX) [file pone.0198783.s004.docx]

| **Gem** |  |  |  |  |  |  |  |  |
| --- | --- | --- | --- | --- | --- | --- | --- | --- |
| **Partners' age (years)** | **10+ years younger** | **5-10 years younger** | **<5 years younger** | **same age** | **<5 years older** | **5-10 years older** | **10+ years older** | **TOTAL** |
| **Male respondents' age (years)** |  |  |  |  |  |  |  |  |
| **10-14** |  |  |  |  |  |  |  |  |
| **15-19** | 0.3 | 0.7 | 41.0 | 50.0 | 7.7 | 0.0 | 0.3 | 300 |
| **20-24** | 0.1 | 3.8 | 56.6 | 31.6 | 6.9 | 1.0 | 0.0 | 710 |
| **25-29** | 1.6 | 17.1 | 49.0 | 23.8 | 6.9 | 1.6 | 0.0 | 563 |
| **30-34** | 3.9 | 25.1 | 40.5 | 24.6 | 4.6 | 0.7 | 0.5 | 410 |
| **35-39** | 11.2 | 27.2 | 35.8 | 16.3 | 7.1 | 1.5 | 0.9 | 338 |
| **40-44** | 10.1 | 31.2 | 33.5 | 19.7 | 2.8 | 1.8 | 0.9 | 218 |
| **45-49** | 15.1 | 29.7 | 25.1 | 20.6 | 4.1 | 2.7 | 2.7 | 219 |
| **50-54** | 20.5 | 24.2 | 33.0 | 15.8 | 2.8 | 0.9 | 2.8 | 215 |
| **55-59** | 23.1 | 31.1 | 25.3 | 17.3 | 1.3 | 1.3 | 0.4 | 225 |
| **60-64** | 18.3 | 27.2 | 29.6 | 21.6 | 0.5 | 2.8 | 0.0 | 213 |
| **65+** | 27.4 | 26.5 | 20.7 | 19.6 | 1.9 | 1.7 | 2.2 | 362 |
