# Supplementary material for: Who are the male sexual partners of adolescent girls and young women? Comparative analysis of population data in three settings prior to DREAMS roll-out
Source: PLoS One. 2018 Sep 28;13(9):e0198783. doi: 10.1371/journal.pone.0198783 (PMC6161870; doi:10.1371/journal.pone.0198783)
Supplement: S2 Table — Data are row percentages. (DOCX) [file pone.0198783.s005.docx]

| Gem |  |  |  |  |  |  |  |  |
| --- | --- | --- | --- | --- | --- | --- | --- | --- |
| Partners' age (yrs) | 10+ years younger | 5-10 years younger | <5 years younger | same age | <5 years older | 5-10 years older | 10+ years older | TOTAL |
| Female respondents' age (yrs) |  |  |  |  |  |  |  |  |
| 10-14' |  |  |  |  |  |  |  |  |
| 15-19 | 0.0 | 0.3 | 0.8 | 21.9 | 51.6 | 19.5 | 5.9 | 374 |
| 20-24 | 0.1 | 0.1 | 0.8 | 21.7 | 47.0 | 22.1 | 8.1 | 778 |
| 25-29 | 0.0 | 0.5 | 1.1 | 22.0 | 40.4 | 26.9 | 9.2 | 815 |
| 30-34 | 0.0 | 0.2 | 1.9 | 20.6 | 34.3 | 26.3 | 16.8 | 578 |
| 35-39 | 0.0 | 0.2 | 1.6 | 22.9 | 30.8 | 27.2 | 17.3 | 445 |
| 40-44 | 0.3 | 0.9 | 2.0 | 19.3 | 27.0 | 29.8 | 20.7 | 352 |
| 45-49 | 0.7 | 0.0 | 1.6 | 22.2 | 26.8 | 30.4 | 18.3 | 306 |
| 50-54 | 0.4 | 0.8 | 1.3 | 25.4 | 24.6 | 31.3 | 16.3 | 240 |
| 55-59 | 0.6 | 0.0 | 0.6 | 24.4 | 26.3 | 29.4 | 18.8 | 160 |
| 60-64 | 0.0 | 0.0 | 3.8 | 23.8 | 26.3 | 26.3 | 20.0 | 80 |
| 65+ | 0.0 | 1.7 | 1.7 | 15.5 | 25.9 | 46.6 | 8.6 | 58 |
