# Supplementary material for: Who are the male sexual partners of adolescent girls and young women? Comparative analysis of population data in three settings prior to DREAMS roll-out
Source: PLoS One. 2018 Sep 28;13(9):e0198783. doi: 10.1371/journal.pone.0198783 (PMC6161870; doi:10.1371/journal.pone.0198783)
Supplement: S3 Table — Data are row percentages. (DOCX) [file pone.0198783.s006.docx]

| Nairobi |  |  |  |  |
| --- | --- | --- | --- | --- |
|  | Partners' age (yrs) |  |  |  |
| Male respondents' age (yrs) | 10-14 | 15-19 | 20-24 | TOTAL |
| 10-14' | 78.5 | 21.4 | 0.0 | 14 |
| 15-19 | 8.5 | 86.0 | 5.4 | 129 |
| 20-24 | 1.1 | 61.5 | 37.4 | 91 |
