# Supplementary material for: Who are the male sexual partners of adolescent girls and young women? Comparative analysis of population data in three settings prior to DREAMS roll-out
Source: PLoS One. 2018 Sep 28;13(9):e0198783. doi: 10.1371/journal.pone.0198783 (PMC6161870; doi:10.1371/journal.pone.0198783)
Supplement: S4 Table — Data are row percentages. (DOCX) [file pone.0198783.s007.docx]

| Nairobi |  |  |  |  |  |  |
| --- | --- | --- | --- | --- | --- | --- |
|  | Partners' age (yrs) |  |  |  |  |  |
| Female respondents' age (yrs) | 10-14 | 15-19 | 20-24 | 25-29 | 30-34 | TOTAL |
| 10-14' | 40.0 | 40.0 | 20.1 | 0.0 | 0.0 | 14 |
| 15-19 | 12.7 | 33.2 | 40.2 | 12.7 | 1.3 | 129 |
| 20-24 | 9.4 | 18.7 | 41.3 | 29.4 | 1.4 | 91 |
