# Supplementary material for: Who are the male sexual partners of adolescent girls and young women? Comparative analysis of population data in three settings prior to DREAMS roll-out
Source: PLoS One. 2018 Sep 28;13(9):e0198783. doi: 10.1371/journal.pone.0198783 (PMC6161870; doi:10.1371/journal.pone.0198783)
Supplement: S5 Table — Data are row percentages. (DOCX) [file pone.0198783.s008.docx]

| **uMkhanyakude** |  |  |  |  |  |  |  |  |  |  |  |  |  |
| --- | --- | --- | --- | --- | --- | --- | --- | --- | --- | --- | --- | --- | --- |
|  | Female partners' age (yrs) | | | |  |  |  |  |  |  |  |  |  |
|  | 10-14 | 15-19 | 20-24 | 25-29 | 30-34 | 35-39 | 40-44 | 45-49 | 50-54 | 55-59 | 60-64 | 65+ | TOTAL |
| Male respondents' age (yrs) |  |  |  |  |  |  |  |  |  |  |  |  |  |
| 15-19 | 3.8 | 91 | 5.2 | 0.0 | 0.0 | 0.0 | 0.0 | 0.0 | 0.0 | 0.0 | 0.0 | 0.0 | 212 |
| 20-24 | 0.0 | 34.7 | 61.0 | 4.3 | 0.0 | 0.0 | 0.0 | 0.0 | 0.0 | 0.0 | 0.0 | 0.0 | 369 |
| 25-29 | 0.0 | 4.2 | 47.5 | 43.3 | 4.6 | 0.4 | 0.0 | 0.0 | 0.0 | 0.0 | 0.0 | 0.0 | 238 |
| 30-34 | 0.0 | 0.5 | 12.6 | 47.5 | 33.8 | 4.0 | 1.5 | 0.0 | 0.0 | 0.0 | 0.0 | 0.0 | 198 |
| 35-39 | 0.0 | 0.0 | 4.2 | 8.3 | 43.8 | 36.5 | 4.2 | 3.1 | 0.0 | 0.0 | 0.0 | 0.0 | 96 |
| 40-44 | 0.0 | 0.0 | 1.2 | 1.2 | 19.3 | 55.4 | 15.7 | 4.8 | 2.4 | 0.0 | 0.0 | 0.0 | 83 |
| 45-49 | 0.0 | 0.0 | 2.3 | 2.3 | 9.1 | 13.6 | 45.5 | 22.7 | 4.5 | 0.0 | 0.0 | 0.0 | 44 |
| 50-54 | 0.0 | 0.0 | 0.0 | 0.0 | 0.0 | 0.0 | 23.8 | 28.6 | 38.1 | 9.5 | 0.0 | 0.0 | 21 |
| 55-59 | 0.0 | 0.0 | 0.0 | 0.0 | 0.0 | 0.0 | 4.5 | 13.6 | 50.0 | 27.3 | 4.5 | 0.0 | 22 |
| 60-64 | 0.0 | 0.0 | 0.0 | 0.0 | 5.9 | 0.0 | 0.0 | 11.8 | 11.8 | 41.2 | 23.4 | 5.9 | 17 |
| 65+ | 0.0 | 0.0 | 0.0 | 3.2 | 0.0 | 0.0 | 0.0 | 6.5 | 9.7 | 3.2 | 29.0 | 48.4 | 31 |
