# Supplementary material for: Who are the male sexual partners of adolescent girls and young women? Comparative analysis of population data in three settings prior to DREAMS roll-out
Source: PLoS One. 2018 Sep 28;13(9):e0198783. doi: 10.1371/journal.pone.0198783 (PMC6161870; doi:10.1371/journal.pone.0198783)
Supplement: S6 Table — Data are row percentages. (DOCX) [file pone.0198783.s009.docx]

| uMkhanyakude |  |  |  |  |  |  |  |  |  |  |  |  |  |
| --- | --- | --- | --- | --- | --- | --- | --- | --- | --- | --- | --- | --- | --- |
|  | Male partners' age (yrs) | | | |  |  |  |  |  |  |  |  |  |
|  | 10-14 | 15-19 | 20-24 | 25-29 | 30-34 | 35-39 | 40-44 | 45-49 | 50-54 | 55-59 | 60-64 | 65+ | TOTAL |
| Female respondents' age (yrs) |  |  |  |  |  |  |  |  |  |  |  |  |  |
| 15-19 | 0.3 | 32.9 | 57.9 | 7.1 | 1.5 | 0.0 | 0.3 | 0.0 | 0.0 | 0.0 | 0.0 | 0.0 | 392 |
| 20-24 | 0.2 | 0.9 | 43.9 | 44.4 | 8.7 | 1.4 | 0.3 | 0.2 | 0.0 | 0.0 | 0.0 | 0.0 | 642 |
| 25-29 | 0.0 | 0.0 | 2.4 | 42.8 | 41.3 | 10.1 | 2.4 | 0.6 | 0.2 | 0.2 | 0.0 | 0.0 | 537 |
| 30-34 | 0.0 | 0.0 | 0.0 | 3.3 | 40.2 | 36.9 | 14.3 | 2.6 | 1.7 | 0.5 | 0.0 | 0.5 | 420 |
| 35-39 | 0.0 | 0.0 | 0.0 | 0.4 | 1.6 | 34.6 | 43.9 | 13.8 | 3.3 | 2.0 | 0.0 | 0.4 | 246 |
| 40-44 | 0.0 | 0.0 | 0.0 | 0.0 | 0.6 | 1.8 | 42.2 | 31.9 | 16.3 | 3.6 | 3.0 | 0.6 | 166 |
| 45-49 | 0.0 | 0.0 | 0.9 | 0.0 | 0.9 | 0.0 | 3.5 | 19.1 | 52.2 | 12.2 | 6.1 | 5.2 | 115 |
| 50-54 | 0.0 | 0.0 | 0.0 | 0.0 | 0.0 | 0.0 | 0.0 | 0.0 | 34.8 | 41.3 | 15.2 | 8.7 | 46 |
| 55-59 | 0.0 | 0.0 | 0.0 | 0.0 | 0.0 | 0.0 | 3.4 | 0.0 | 3.4 | 37.9 | 41.4 | 13.8 | 29 |
| 60-64 | 0.0 | 0.0 | 0.0 | 0.0 | 0.0 | 0.0 | 0.0 | 0.0 | 0.0 | 0.0 | 52.9 | 47.1 | 17 |
| 65+ | 0.0 | 0.0 | 0.0 | 0.0 | 0.0 | 0.0 | 0.0 | 0.0 | 0.0 | 0.0 | 0.0 | 100 | 13 |
