# Supplementary material for: Who are the male sexual partners of adolescent girls and young women? Comparative analysis of population data in three settings prior to DREAMS roll-out
Source: PLoS One. 2018 Sep 28;13(9):e0198783. doi: 10.1371/journal.pone.0198783 (PMC6161870; doi:10.1371/journal.pone.0198783)
Supplement: S7 Table — (DOCX) [file pone.0198783.s010.docx]

| **Gem** |  |  |  |  |  |  |  | |  |  |  |  |  |
| --- | --- | --- | --- | --- | --- | --- | --- | --- | --- | --- | --- | --- | --- |
|  | **Females** |  |  |  |  |  | **Males** | | |  |  |  |  |
|  | **15-19 years** | | **20-24 years** | | **15-24 years** | | **Report at least one partner aged 15-24 years** | | | **Aged 20-34 years & report at least one partner possibly aged 15-24 years** | | **Aged 35+ years & report at least one partner possibly aged 15-24 years** | |
|  | **n** | **%** | **n** | **%** | **n** | **%** | **n** | **%** | | **n** | **%** | **n** | **%** |
| **Lifetime number of sexual partners (among those who ever had sex)** | | | |  |  |  |  |  | |  |  |  |  |
| median (IQR) |  | **2 (1,2)** |  | **2 (1,3)** |  |  |  | **3 (2,8)** | |  | **5 (3,9)** |  | **9 (5,9)** |
| 1 | 235 | **46.9** | 255 | **27.4** | 490 | **34.2** | 85 | **16.1** | | 45 | **8.3** | 12 | **4.0** |
| 2 | 147 | **29.3** | 316 | **33.9** | 463 | **32.3** | 106 | **20.0** | | 69 | **12.7** | 17 | **5.7** |
| 3 | 75 | **15.0** | 194 | **20.8** | 269 | **18.8** | 81 | **15.3** | | 97 | **17.8** | 22 | **7.4** |
| 4 | 24 | **4.8** | 58 | **6.2** | 82 | **5.7** | 57 | **10.8** | | 51 | **9.4** | 20 | **6.7** |
| 5+ | 14 | **2.8** | 64 | **6.9** | 78 | **5.4** | 132 | **25.0** | | 176 | **32.3** | 109 | **36.6** |
| Don't know | 6 | **1.2** | 44 | **4.7** | 50 | **3.5** | 68 | **12.9** | | 107 | **19.6** | 118 | **39.6** |
| **Type of partners in past 12 months** |  |  |  |  |  |  |  |  | |  |  |  |  |
| Single spouse | 183 | **49.3** | 613 | **76.9** | 796 | **68.2** | 81 | **15.4** | | 278 | **50.6** | 184 | **61.7** |
| Single regular partner | 157 | **42.3** | 155 | **19.5** | 312 | **26.7** | 292 | **55.4** | | 133 | **24.2** | 4 | **1.3** |
| Single casual partner | 3 | **0.8** | 4 | **0.5** | 7 | **0.6** | 4 | **0.8** | | 0 | **0.0** | 5 | **1.7** |
| Multiple partners- spouse/regular | 24 | **6.5** | 23 | **2.9** | 47 | **4.0** | 136 | **25.8** | | 120 | **21.8** | 88 | **29.5** |
| Multiple partners- casual | 0 | **0.0** | 0 | **0.0** | 0 | **0.0** | 1 | **0.2** | | 1 | **0.2** | 0 | **0.0** |
| Multiple partners-spouse/regular and casual | 4 | **1.1** | 2 | **0.3** | 6 | **0.5** | 13 | **2.5** | | 18 | **3.3** | 17 | **5.7** |
| **Type of concurrent partners** |  |  |  |  |  |  |  |  | |  |  |  |  |
| Spouse/regular | 7 | **87.5** | 9 | **100** | 16 | **94.1** | 65 | **98.5** | | 60 | **95.2** | 80 | **86.0** |
| Casual | 0 | **0.0** | 0 | **0.0** | 0 | **0.0** | 0 | **0.0** | | 1 | **1.6** | 0 | **0.0** |
| Spouse/regular & casual | 1 | **12.5** | 0 | **0.0** | 1 | **5.9** | 1 | **1.5** | | 2 | **3.2** | 13 | **14.0** |

| **Nairobi** |  |  |  |  |  |  |  |  |  |  |  |  |
| --- | --- | --- | --- | --- | --- | --- | --- | --- | --- | --- | --- | --- |
|  | **Females** |  |  |  |  |  | **Males** | |  |  |  |  |
|  | **15-19 yrs** | | **20-24 yrs** | | **15-24 yrs** | | **Report partner aged 15-19 yrs** | | **Report partner aged 20-24 yrs** | | **Report partner aged 15-24 yrs** | |
|  | n | % | **n** | **%** | **n** | **%** | n | % | **n** | **%** | **n** | **%** |
| **Lifetime number of sexual partners (among those who ever had sex)** | | | |  |  |  |  |  |  |  |  |  |
| median (IQR) |  | **1 (1,2)** | **1 (1,2)** | |  |  | **2 (1,4)** | | **3 (1,5)** | | **3 (1,4)** | |
| 1 | 85 | **70.3** | 195 | **54.6** | 280 | **58.6** | 53 | **36.6** | 15 | **38.5** | 68 | **37.0** |
| 2 | 18 | **14.9** | 83 | **23.3** | 101 | **21.1** | 36 | **24.8** | 4 | **10.3** | 40 | **21.7** |
| 3 | 9 | **7.4** | 41 | **11.5** | 50 | **10.5** | 19 | **13.1** | 3 | **7.7** | 22 | **12.0** |
| 4 | 2 | **1.7** | 10 | **2.8** | 12 | **2.5** | 20 | **13.8** | 3 | **7.7** | 23 | **12.5** |
| 5+ | 3 | **2.5** | 18 | **5.0** | 21 | **4.4** | 17 | **11.7** | 14 | **35.9** | 31 | **16.8** |
| Missing | 4 | **3.3** | 10 | **2.8** | 14 | **2.9** |  |  |  |  |  |  |

| **uMkhanyakude** |  |  |  |  |  |  |  |  |  |  |  |  |  |
| --- | --- | --- | --- | --- | --- | --- | --- | --- | --- | --- | --- | --- | --- |
|  | **Females** |  |  |  |  |  | **Males** | |  |  |  |  |  |
|  | **15-19 yrs** | | **20-24 yrs** | | **15-24 yrs** | | **Report partner aged 15-19 yrs** | | **Report partner aged 20-24 yrs** | | **Report partner aged 15-24 yrs** | | |
| **Lifetime number of sexual partners (among those who ever had sex)** | | | |  |  |  |  |  |  |  |  |  |  |
| median (IQR) |  | **1 (1,1)** |  | **1(1,2)** |  |  |  | **2(1,3)** |  | **3(1,4)** |  | **2(1,3)** |  |
| 1 | 340 | **80.4** | 372 | **54.9** | 712 | **64.7** | 129 | **39.6** | 92 | **24.7** | 221 | **31.7** |  |
| 2 | 54 | **12.8** | 165 | **24.3** | 219 | **19.9** | 60 | **18.4** | 46 | **12.3** | 106 | **15.2** |  |
| 3 | 18 | **4.3** | 80 | **11.8** | 98 | **8.9** | 51 | **15.6** | 60 | **16.1** | 111 | **15.9** |  |
| 4 | 2 | **0.5** | 23 | **3.4** | 25 | **2.3** | 31 | **9.5** | 40 | **10.7** | 70 | **10.0** |  |
| 5+ | 2 | **0.5** | 11 | **1.6** | 13 | **1.2** | 19 | **5.8** | 47 | **12.6** | 65 | **9.3** |  |
| Don't know | 7 | **1.7** | 27 | **4.0** | 34 | **3.1** | 36 | **11.0** | 88 | **23.6** | 124 | **17.8** |  |
| **Type of partners in past 12 months** |  |  |  |  |  |  |  |  |  |  |  |  |  |
| Single spouse | 0 | **0.0** | 7 | **1.0** | 7 | **0.6** | 0 | **0.0** | 0 | **0.0** | 0 | **0.0** |  |
| Single regular partner | 366 | **89.3** | 603 | **90.1** | 969 | **89.8** | 250 | **77.4** | 327 | **89.1** | 577 | **83.9** |  |
| Single casual partner | 40 | **9.8** | 53 | **7.9** | 93 | **8.6** | 67 | **20.7** | 30 | **8.2** | 97 | **14.1** |  |
| Multiple partners- spouse/regular | 3 | **0.7** | 3 | **0.4** | 6 | **0.6** | 0 | **0.0** | 2 | **0.5** | 2 | **0.3** |  |
| Multiple partners- casual | 0 | **0.0** | 0 | **0.0** | 0 | **0.0** | 3 | **0.9** | 4 | **1.1** | 6 | **0.9** |  |
| Multiple partners-spouse/regular and casual | 1 | **0.2** | 3 | **0.4** | 4 | **0.4** | 3 | **0.9** | 4 | **1.1** | 6 | **0.9** |  |
| **Type of concurrent partners** |  |  |  |  |  |  |  |  |  |  |  |  |  |
| Spouse/regular | 2 | **66.7** | 2 | **50.0** | **4** | **57.1** | 0 | **0.0** | 2 | **25.0** | 2 | **18.2** |  |
| Casual | 0 | **0.0** | 0 | **0.0** | **0** | **0.0** | 2 | **40.0** | 3 | **37.5** | 4 | **36.4** |  |
| Spouse/regular & casual | 1 | **33.3** | 2 | **50.0** | **3** | **42.9** | 3 | **60.0** | 3 | **37.5** | 5 | **45.5** |  |
