# Supplementary material for: Who are the male sexual partners of adolescent girls and young women? Comparative analysis of population data in three settings prior to DREAMS roll-out
Source: PLoS One. 2018 Sep 28;13(9):e0198783. doi: 10.1371/journal.pone.0198783 (PMC6161870; doi:10.1371/journal.pone.0198783)
Supplement: S8 Table — (DOCX) [file pone.0198783.s011.docx]

| **Gem** |  |  |  |  |  |  |
| --- | --- | --- | --- | --- | --- | --- |
|  | **Type of partner** | |  |  |  |  |
|  | **Spousal/ co-resident partner** | | **Other regular** | | **Casual** | |
|  | **n** | **%** | **n** | **%** | **n** | **%** |
|  | *801* | *66.4* | *394* | *32.7* | *11* | *0.9* |
| **How respondent found out partner's HIV status** |  |  |  |  |  |  |
| Partner told me | 94 | 16.9 | 46 | 18.6 | 3 | 75.0 |
| We tested together | 444 | 79.7 | 199 | 80.6 | 1 | 25.0 |
| Other (I saw/heard about the results another way, I just know, refuse to answer) | 19 | 3.4 | 2 | 0.8 | 0 | 0.0 |
| **Partner acquired new partners in the past year** |  |  |  |  |  |  |
| Yes | 90 | 11.2 | 54 | 13.7 | 2 | 18.2 |
| I think so | 27 | 3.4 | 25 | 6.4 | 2 | 18.2 |
| No | 381 | 47.6 | 134 | 34.0 | 3 | 27.3 |
| Don't know | 302 | 37.7 | 179 | 45.4 | 4 | 36.4 |
| Refuse to answer/ not applicable | 1 | 0.1 | 2 | 0.6 | 0 | 0.0 |
| **Ever received money/gifts for each partner** |  |  |  |  |  |  |
| Yes, everytime | 0 | 0.0 | 70 | 17.8 | 3 | 27.3 |
| Yes, sometimes or occasionally | 0 | 0.0 | 150 | 38.1 | 3 | 27.3 |
| No, never | 0 | 0.0 | 172 | 43.7 | 5 | 45.5 |
| Refuse to answer | 0 | 0.0 | 2 | 0.5 | 0 | 0.0 |
| Not applicable | 778 | 100.0 | 0 | 0.0 | 0 | 0.0 |
|  |  |  |  |  |  |  |
| **Ever given money/gifts for each partner** |  |  |  |  |  |  |
| Yes, everytime | 0 | 0.0 | 8 | 2.0 | 0 | 0.0 |
| Yes, sometimes or occasionally | 0 | 0.0 | 42 | 10.7 | 1 | 9.1 |
| No, never | 0 | 0.0 | 343 | 87.1 | 10 | 90.9 |
| Refuse to answer | 0 | 0.0 | 1 | 0.3 | 0 | 0.0 |
| Not applicable | 778 | 100.0 | 0 | 0.0 | 0 | 0.0 |
| **Forced by partner to have sex in past 12 month** |  |  |  |  |  |  |
| Yes | 75 | 9.4 | 23 | 5.8 | 1 | 9.1 |
| No | 726 | 90.6 | 370 | 93.9 | 10 | 90.9 |
| Don't know | 0 | 0.0 | 1 | 0.3 | 0 | 0.0 |
|  |  |  |  |  |  |  |
